# A New Anti-Depressive Strategy for the Elderly: Ablation of FKBP5/FKBP51

**DOI:** 10.1371/journal.pone.0024840

**Published:** 2011-09-15

**Authors:** John C. O'Leary, Sheetal Dharia, Laura J. Blair, Sarah Brady, Amelia G. Johnson, Melinda Peters, Joyce Cheung-Flynn, Marc B. Cox, Gabriel de Erausquin, Edwin J. Weeber, Umesh K. Jinwal, Chad A. Dickey

**Affiliations:** 1 Department of Molecular Medicine, Byrd Alzheimer's Research Institute, University of South Florida, Tampa, Florida, United States of America; 2 Department of Physiology and Pharmacology, Byrd Alzheimer's Research Institute, University of South Florida, Tampa, Florida, United States of America; 3 Department of Surgery, Vanderbilt University Medical Center, Nashville, Tennessee, United States of America; 4 Department of Biological Sciences, University of Texas at El Paso, El Paso, Texas, United States of America; 5 Border Biomedical Research Center, University of Texas at El Paso, El Paso, Texas, United States of America; 6 College of Pharmacy, Byrd Alzheimer's Research Institute, University of South Florida, Tampa, Florida, United States of America; 7 Department of Psychiatry and Neuroscience, University of South Florida, Tampa, Florida, United States of America; University of Chicago, United States of America

## Abstract

The gene *FKBP5* codes for FKBP51, a co-chaperone protein of the Hsp90 complex that increases with age. Through its association with Hsp90, FKBP51 regulates the glucocorticoid receptor (GR). Single nucleotide polymorphisms (SNPs) in the *FKBP5* gene associate with increased recurrence of depressive episodes, increased susceptibility to post-traumatic stress disorder, bipolar disorder, attempt of suicide, and major depressive disorder in HIV patients. Variation in one of these SNPs correlates with increased levels of FKBP51. FKBP51 is also increased in HIV patients. Moreover, increases in FKBP51 in the amygdala produce an anxiety phenotype in mice. Therefore, we tested the behavioral consequences of *FKBP5* deletion in aged mice. Similar to that of naïve animals treated with classical antidepressants *FKBP5*−/− mice showed antidepressant behavior without affecting cognition and other basic motor functions. Reduced corticosterone levels following stress accompanied these observed effects on depression. Age-dependent anxiety was also modulated by *FKBP5* deletion. Therefore, drug discovery efforts focused on depleting FKBP51 levels may yield novel antidepressant therapies.

## Introduction

Genes regulating the hypothalamus-pituitary-adrenal (HPA) axis are associated with susceptibility to depression as well as antidepressant efficacy [1], [2], [3]. The HPA axis has a well-characterized role as a regulator of the neuroendocrine stress response [4]. Its activation leads to the production of glucocorticoids in the adrenal axis, of which the major constituent in humans is cortisol and in rodents is corticosterone. Over the past decade, genome wide association studies for single nucleotide polymorphisms (SNPs) revealed significant associations between susceptibility to depressive episodes and variants in both the *NR3C1*, that encodes the glucocorticoid receptor (GR), and *FKBP5*, that encodes a GR binding protein thought to attenuate GR activity [2], [5]. While most studies have focused on the variants in GR because of its role as a transcriptional regulator [6], the involvement of *FKBP5* and its gene product, FKBP51, have received little attention. This is largely due to uncertainty about how to approach this relatively unknown protein. In fact, it remains to be proven whether FKBP51 is a valid therapeutic target for treating depression, despite its clear genetic link.

Since the initial discovery of the association between *FKBP5* SNPs and depression, other psychiatric disorders have been found to be associated with *FKBP5* SNPs including PTSD [7], bipolar disorder [8], anxiety [9], peritraumatic dissociation [10], and major depression in HIV patients [11]. The TT variant of the rs1360780 SNP was associated with both an increased incidence of depressive episodes throughout a carrier's lifetime, and increased sensitivity to common neurotransmitter-based anti-depressants [2]. Interestingly, individuals with the rs1360780 TT *SNP* had significantly higher FKBP51 protein levels in their lymphocytes. FKBP51 levels are also elevated in patients with HIV infection, perhaps playing a role in the depression that commonly occurs with chronic highly active antiretroviral therapies (HAART) [12]. Recently, stress was shown to induce neuropsin activity in the amygdala, inducing anxiety in mice through an FKBP51-dependent mechanism [13]. How FKBP51 directly modulates GR has been investigated *in vitro*. In these systems, upregulation of FKBP51 decreases the affinity of GR for its substrate. This in turn decreases the amount of GR that becomes transcriptionally active [9]. In new world monkeys a naturally occurring glucocorticoid resistance has been attributed to higher than normal levels of FKBP51. However, reduced GR activity in a transgenic mouse model of FKBP51 overexpression has never been shown [14], [15].

While the causes of major depressive disorders are unknown, there is an emerging genetic diathesis for its occurrence within genes regulating the HPA axis; however few animal models have been developed or utilized for aetiologic validation studies. Genetic variation in FKBP51 appears to be one factor that facilitates liability to anxiety and mood disorders. Thus, the goal of this study was to determine whether decreasing FKBP51 expression could make mice less susceptible to inducible “depression-like” states through a corticosterone-dependent mechanism *in vivo* in well established models with high predictive value [16]. Indeed, aged *FKBP5*-deficient mice were resistant to stress-induced depressive-like behavior. Moreover, despite robust hippocampal and forebrain expression patterns, deletion of *FKBP5* did not result in cognitive impairment or other behavioral abnormalities. Circulating levels of corticosterone in the same *FKBP5−/−* mice were also reduced after stress, confirming the proposed mechanism previously described [9]. These data suggest that not only is FKBP51 a valid therapeutic target, but targeting this protein may also have minimal consequences for other behavioral characteristics.

## Results

### FKBP5−/− mice

**Figure 1:**
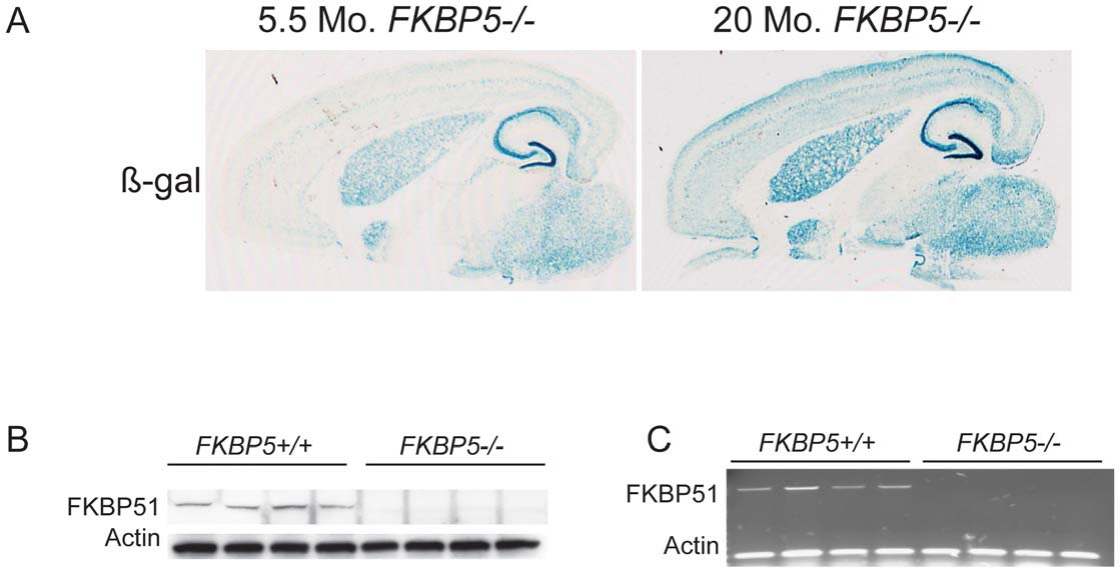
Distribution and confirmation of gene knockout. (A) Representative images of β-gal staining in horizontal brain slices of 5.5 and 20 month old *FKBP5−/−* mice. (B) Western blot analysis of 20-month old wildtype and FKBP5−/− whole brain homogenates. (C) *FKBP5* primer-specific PCR of cDNA synthesized from brain-isolated mRNA by reverse transcription.

*FKBP5−/−* mice were used to determine the effect of gene deletion on behavior. The mice contain a β-galactosidase reporter cassette, which expresses wherever the *FKBP5* gene is normally expressed. To confirm gene knockout and establish cerebral distribution of FKBP51, tissue from 5.5 and 20-month-old *FKBP5*−/− mice was stained using an X-gal kit that produces a blue product when β-galactosidase is present. An age-dependent increase in β-gal expression was observed particularly in the upper cortical layers of the forebrain (Figure 1A). This was consistent with the age-dependent increase in FKBP51 expression that had been previously reported in normal mice [17]. To confirm the absence of FKBP51 protein, whole brain homogenates were analyzed by immunoblot and probed using an FKBP51 antibody. No detectable FKBP51 was observed (Figure 1B). To verify that FKBP51 mRNA was absent, oligo d(T) RT-PCR was performed using reverse transcriptase to produce cDNA. No *FKBP5* PCR product was detected in the *FKBP5*−/− mice, confirming that no FKBP51 mRNA was present (Figure 1C).

### Antidepressive behavior in FKBP5−/− mice

**Figure 2:**
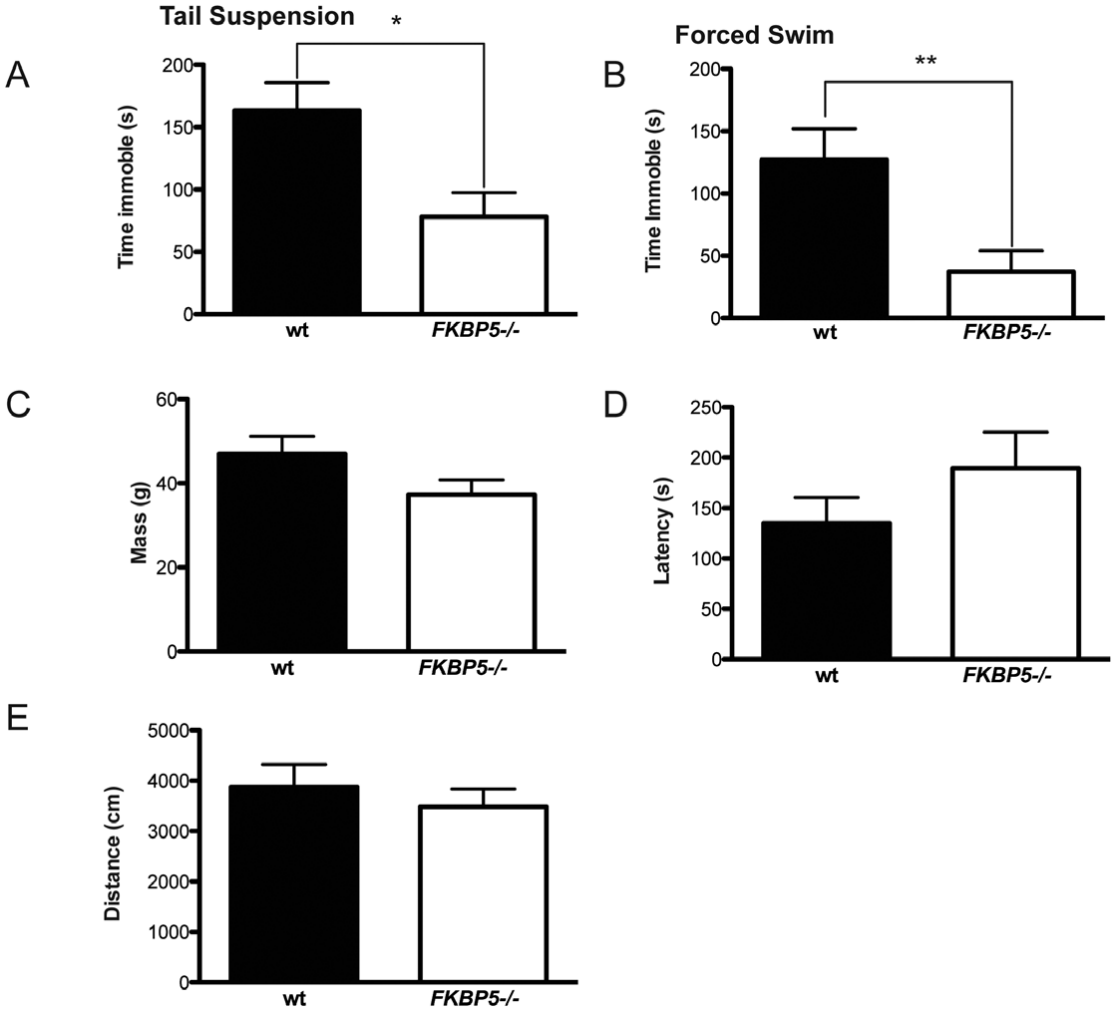
*FKBP5−/−* mice display resistance to depression inducing stimulus. (A) Total time immobile in the tail suspension test; *p<0.05, t = 2.870, df = 16. (B) Total time immobile in the forced swim test; **p<0.01, t = 2.982, df = 16. (C) No significant difference was observed between mass measurements of groups. (D) Latency to fall from a gradually accelerating rotorod apparatus displays no difference between groups. (E) Activity levels in the open field display no differences between groups.

*FKBP5−/−* mice aged 17–20 months were submitted to two behavioral models of depressive-like activity; the classical forced swim test (FST) and the tail suspension test (TST) [18], [19]. Results from these tests are based on the total time spent immobile over a 6-minute period, interpreted as despair. These tests are typical for assessing antidepressant efficacy [18]. Aged *FKBP5−/−* mice displayed a shorter immobility time than their wildtype counterparts (Figure 2A–2B). The weight, activity, and physical fitness of the mice were evaluated to account for possible confounding factors, but these variables did not contribute to the effect (Figure 2C–2E).

### Effect of FKBP5−/− on corticosterone production

**Figure 3:**
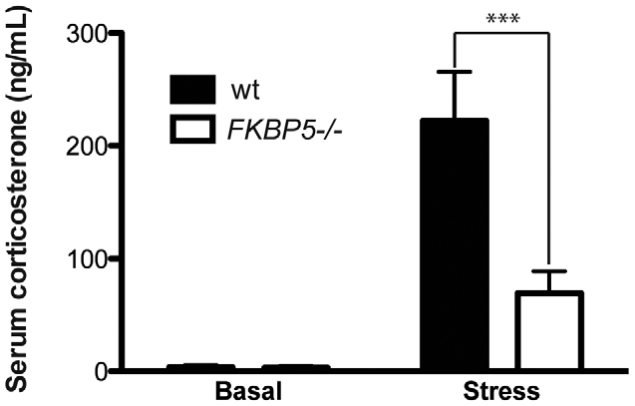
Effects of *FKBP5−/−* on corticosterone production. Levels of corticosterone in serum right before a 10-minute tube restraint and 30 minute after (wt n = 5, *FKBP5−/−* n = 6); interaction F(1,18) = 11.54, **p<0.01; genotype F(1,18) = 11.72, **p<0.01; stress F(1,18) = 40.5, ***p<0.0001; Bonferroni multiple comparisons wt vs *FKBP5−/−* stress, t = 4.82, ***p<0.001.

Stress causes increased levels of cortisol in humans and depressed patients have higher-than-normal levels of cortisol in the blood [20]. The dexamethasone-corticotropin releasing hormone (DEX-CRH) test, a commonly used tool to detect HPA system changes, has been used to link depressive behavior with glucocorticoid receptor (GR) insensitivity [21]. To determine if *FKBP5* deletion was altering glucocorticoid levels, circulating corticosterone levels were assessed in the same aged cohort of *FKBP5−/−* mice that were used for behavioral analyses. Early morning blood draws were collected from both *FKBP5−/−* and wildtype littermates before and 30 minutes after being placed in a restrainer for 10 minutes. Levels of basal corticosterone were predictably low, consistent with previous reports showing low levels of corticosterone production in the earliest part of the murine diurnal cycle (Figure 3) [22]. After stress corticosterone levels rose in both wildtype and *FKBP5−/−* mice, but this induction was attenuated in the *FKBP5−/−* mice. These findings suggest that the lack of FKBP51 permits unrestrained GR transcription activity, which decreases HPA-axis activity and corticosterone levels, improving resilience to depressive-like behavior.

### Characterization of anxious behavior in the FKBP5−/− model

**Figure 4:**
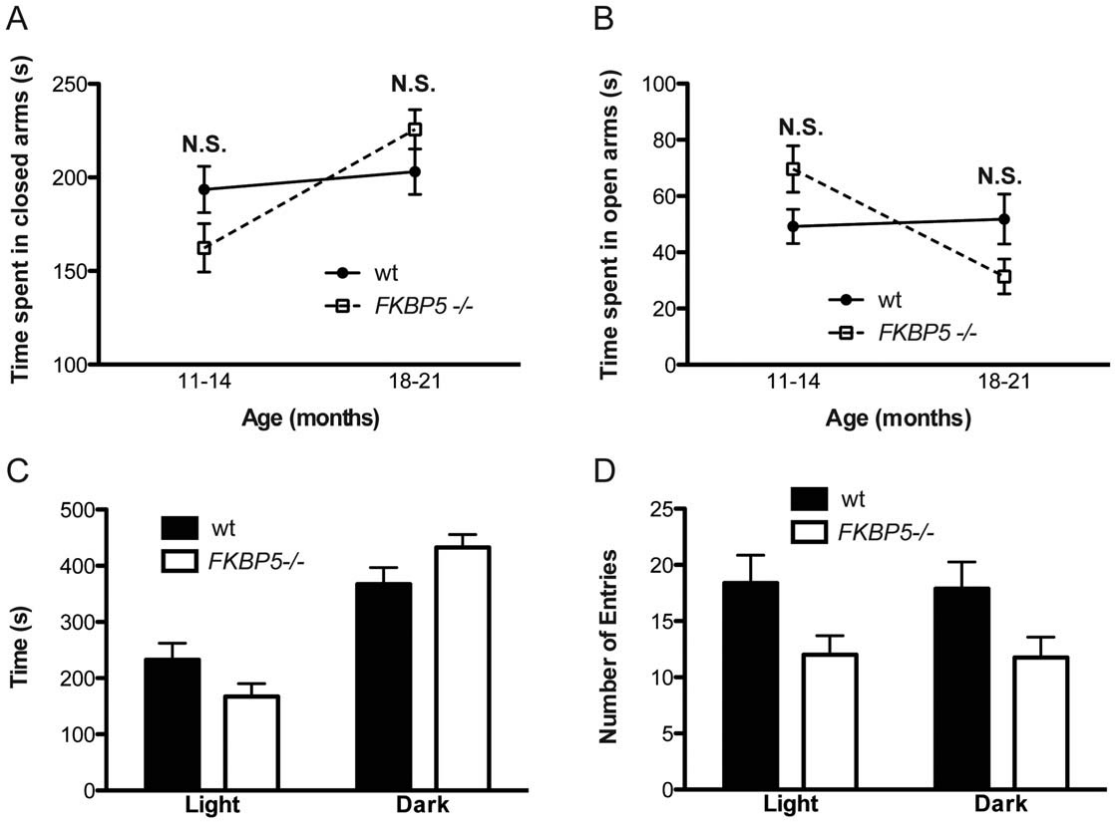
Age-associated changes in anxious behavior in *FKBP5−/−* mice. (A) Time spent in closed arms of the EPM (n = 8 for each group); interaction F(1,14) = 4.59, p = 0.0502; genotype F(1,14) = 0.14, p = 0.713; time F(1,14) = 8.38, *p<0.05. (B) Time spent in open arms of the EPM (n = 8 for each group); interaction F(1,14) = 8.30, *p<0.05; genotype F(1,14) = 0.0, p = 0.999; time F(1,14) = 6.33, *p<0.05. (C) Time spent in the areas of the dark-light chamber (wt n = 8, *FKBP5−/−* n = 8); interaction F(1,28) = 6.13, *p<0.05; genotype F(1,28) = 0, p = 0.999; amount of light in chamber F(1,28) = 57.9, ***p<0.0001. (D) Number of entries into the areas of the dark-light chamber (wt n = 8, *FKBP5−/−* n = 8); interaction F(1,26) = 0.01, p = 0.933; genotype F(1,26) = 5.82, *p<0.05; amount of light in chamber F(1,26) = 0.02, p = 0.879.

Brain-specific knockout of GR in mice reduces anxiety [23]. Also, FKBP51 expression was recently correlated to anxious behavior [13]. Based on this and the novel link now established between FKBP51, GR activity and glucocorticoid production, the effects of *FKBP5* deletion on anxiety were tested. The longitudinal impact of *FKBP5* deletion on anxiety was assessed in mice aged 11–14 months and then again in the same mice aged 18–22 months using the elevated plus maze (EPM). A repeated measures two-way analysis of variance was conducted to examine the effect of time and genotype on anxiety. The behavioral correlates of anxiety in the EPM were significantly affected by age, and this change depended on the genotype at *FKBP5* as reflected by a significant age*genotype interaction, (Figure 4A–4B). Indeed, in wildtype mice behavioral correlates of anxiety decreased with age, whereas they appeared to increase in *FKBP5*−/− mice. To further examine whether anxiety was indeed being modified in these mice, the 18–22 month-old mice were subjected to the light-dark chamber paradigm, which is another standard measure of anxiety. The mice were allowed to explore between a well-lit and unprotected area and a dark covered area that the mice had access to through a small opening. Normal mice typically move to the dark chamber. The light-dark maze did not reveal any statistical differences between the groups (Figure 4C–4D). These data suggest that suppression of FKBP51 may have a differential effect on anxiety depending on the developmental stage; however, the effect is not pervasive enough to manifest itself through several indices of anxiety.

### Memory and general behavioral characterization of FKBP5−/− mice

**Figure 5:**
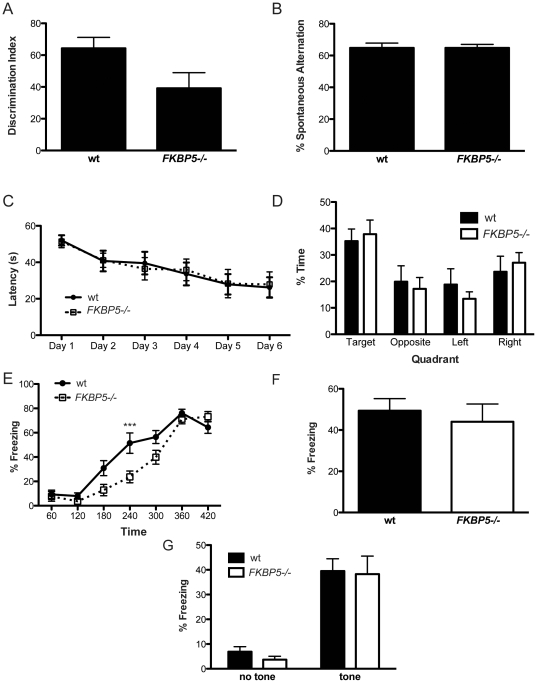
Deletion of the FKBP5 gene does not alter learning and memory despite robust expression of FKBP5 in the hippocampus. (A) Discrimination index between familiar and novel object in the novel object recognition test. (B) Percent of spontaneous alternation shows no difference between the groups. (C) Learning of the location of the hidden platform in the MWM (interaction F(5,80) = 0.14, p = 0.98; genotype F(1,16) = 0.0, p = 0.98; time F(5,80) = 12.71, p<0.0001). (D) Percent time spent in quadrant during the probe trial of MWM. (E) Software generated heat plot representing amount of time spent in an area. (F) Percent freezing during training portion of fear conditioning; interaction F(6,90) = 4.1, **p = 0.001; genotype F(1,15) = 5.66, *p = 0.031; time F(6,90) = 79.89, ***p<0.0001; Bonferroni multiple comparisons wt vs *FKBP5−/−* at 240 s, t = 2.42, ***p<0.001. (G) Percent freezing during contextual exposure to fear-conditioning chamber. (H) Percent freezing during the cued fear-conditioning test; interaction F(1,30) = 0.05, p = 0.828; genotype F(1,30) = 0.25, p = 0.624; tone F(1,30) = 55.75, ***p<0.0001.

Untoward consequences of FKBP51 ablation were a distinct possibility given the ubiquitous expression of FKBP51 throughout the brain. Given the high levels of expression of *FKBP5* in the hippocampus (Figure 1A) memory formation was assessed. Therefore, short-term memory in the *FKBP5−/−* mice was tested with the use of the novel object recognition and Y-maze paradigms. Both tests are performed in a short period of time to capture the working memory ability of the mice. Neither test showed statistically significant differences between *FKBP5−/−* and wildtype littermates (Figure 5A–5B). Long-term spatial memory function was then tested using the Morris water maze (MWM). Memory retention was tested 24 hours after the training was completed. The training phase of the MWM displayed that wildtype and *FKBP5*−/− mice were equally capable of learning the location of a hidden platform (Figure 5C). Moreover wildtype and *FKBP5−/−* mice displayed an equivalent capacity to locate the hidden platform 24 hours after training (Figure 5D).

**Figure 6:**
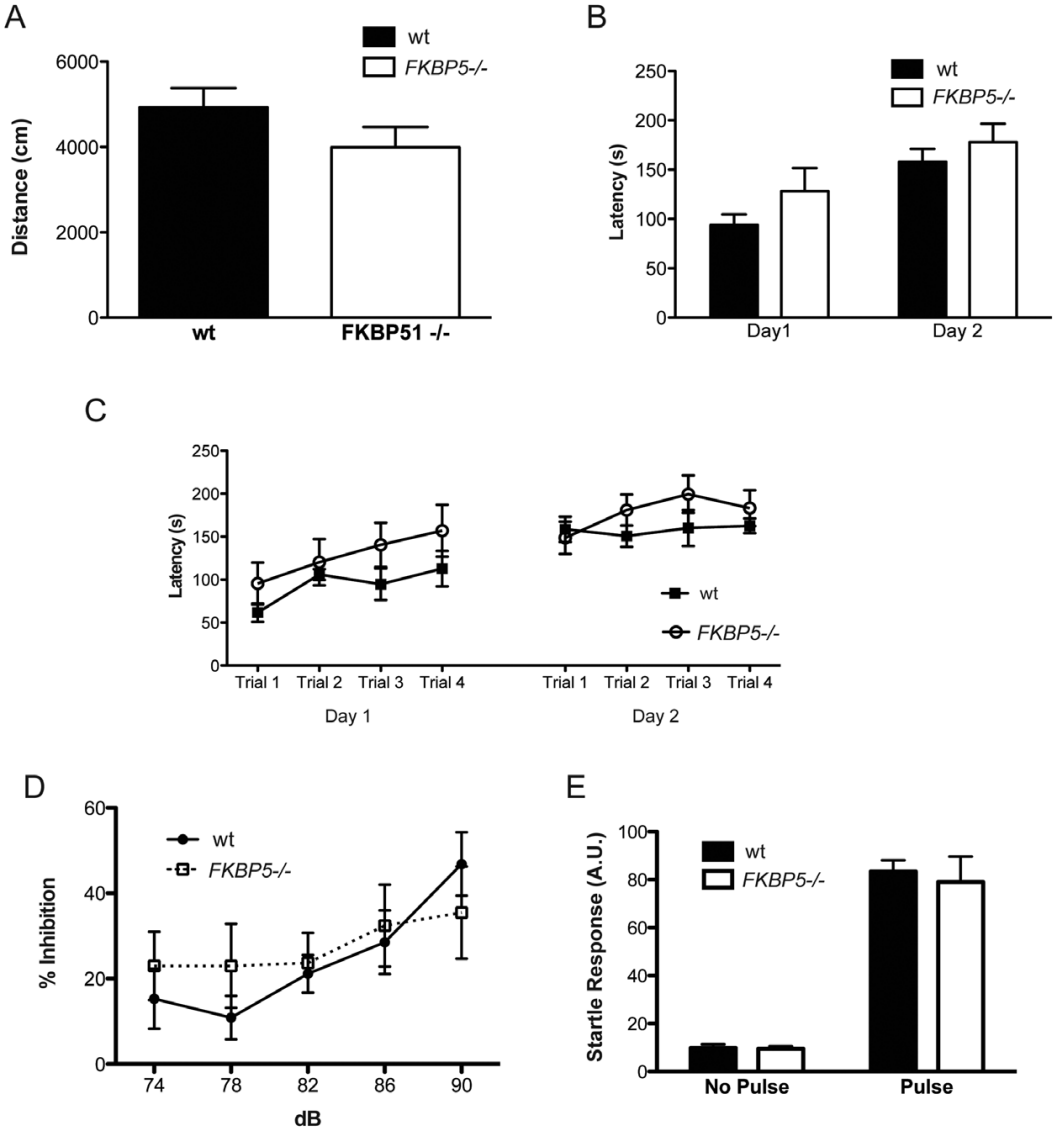
Behavioral characterization of *FKBP5−/−* mice. (A) Distance traveled in the open field test. (B) Assessment of motor learning from comparison between the average of all four trials of rotorod from day one with those of day two; interaction F(1,16) = 1.28, p = .275; genotype F(1,16) = 1.37, p = 0.259; time F(1,16) = 80.3, ***p<0.0001. (C) Latency to fall from the rotorod apparatus; interacton F(7,112) = 1.28, p = 0.267; genotype F(1,16) = 1.37, p = 0.259; time F(7,112) = 17.97, ***p<0.0001. (D) Percent inhibition of startle response by increasing prepulse intensity; interaction F(4,70) = 0.61, p = 0.656; genotype F(1,70) = 0.33,p = 0.565; prepulse intensity F(4,70) = 3.07, *p<0.05. (E) Comparison of no pulse (no tone) with a 120 dB pulse (tone) to test hearing; interaction F(1,32) = 0.11, p = 0.74; genotype F(1,32) = 0.16, p = 0.694; pulse F(1,32) = 147.58, ***p<0.0001.

The amygdala controls emotional learning in the brain and *FKBP5* expression can be upregulated by GR activation [24]. As a consequence, function of the amygdala could be affected by *FKBP5* deletion. To test the impact of *FKBP5−/−* on emotionally derived memory function, a fear-conditioning paradigm was employed. In this test, a tone played for 30 seconds is followed by a small foot shock. Thus the animal learns to associate the tone with the shock. The amygdala-associated fear response caused by the tone is able to bypass the hippocampus once the association is made, allowing for assessment of emotionally driven memory formation. Surprisingly, no differences between wildtype and *FKBP5−/−* mice were observed in either contextual (environmentally-based) or cued (tone-based) fear conditioning paradigms (Figure 5E–5G). These findings suggest that neither spatially nor emotionally driven long-term memory is affected by *FKBP5* deletion. In addition to these learning and memory tasks, no observable differences between wildtype and *FKBP5−/−* mice were noted in tasks designed to assess activity, motor performance, motor coordination, motor learning, hearing, or prepulse inhibition, which measures startle response inhibition (Figure 6).

## Discussion

Major depression is a devastating disease with a course that is frequently chronic or recurrent and affects millions of people. Research in the last decade has shown that variation in the *FKBP5* gene is associated with depression and several other mood and anxiety disorders. And although *in vitro* data suggests the possibility of a causal relationship between FKBP5 expression levels and depression, this has never been tested *in vivo*. Here we show for the first time that ablation of *FKBP5* in mice led to reduced immobility in two behavioral models that are routinely used to assess anti-depressant efficacy. This behavioral effect coincided with attenuation of corticosterone production after a stressful episode. Moreover, no defects in locomotion, somato-sensation or learning and memory were observed. Thus, therapies developed to reduce FKBP51 levels may be highly efficacious as next generation anti-depressants. Furthermore, because FKBP51 ablation results in reduced anxiety-like behavior in mice [13], we provide experimental support for the notion that genetically-driven variations in expression of FKBP51 may underlie susceptibility to anxiety and mood disorders, as suggested by association studies in humans [2], [9].

FKBP51 is a peptidyl-prolyl cis-trans isomerase (PPIase) enzyme that also associates with the chaperone Hsp90 and is distributed ubiquitously throughout the brain. This isomerase activity is thought to be important for structural rearrangements and phosphorylation dynamics of client proteins bound by Hsp90. Several diseases in addition to psychiatric conditions have implicated FKBP51 as having a role in their pathogenesis. These include prostate cancer [25], and neurodegenerative diseases, specifically tauopathies [17]. In fact, these effects on tau may somehow be tied to the manifestation of these psychiatric conditions. Indeed, depleting FKBP51 levels was shown to also reduce tau levels, while inhibiting its PPIase activity actually lead to increased stability of phosphorylated tau. Thus it is certainly possible that FKBP51 is involved in Alzheimer's disease progression, since one of its earliest clinical features is depression. More recently, the extracellular protease neuropsin was shown to mediate anxiety-like behavior via an FKBP51 dependent mechanism [13]. Thus, an important role for FKBP51 in maintaining proper brain function is emerging. Its relationship with major depressive disorder in HIV, bipolar disorder and possibly anxiety and Alzheimer's disease further underlie its significance.

Current treatment for depression includes the use of medications that extend the amount of time neurotransmitters are present in the synaptic cleft including serotonin, norepinephrine, and dopamine. It is estimated that 60–70% of patients reach remission with the use of anti-depressant drugs [26]. These low rates of efficacy have prompted research into other potential therapeutic targets in the HPA axis, particularly GR. However, there are many different isoforms of GR, making selective targeting with compounds challenging. Therefore, the results presented here show that FKBP51 may be the most appropriate target for treating depression via the modulation of the HPA axis in terms of its risk/benefit equation and potential therapeutic window. Also, and most noteworthy, because FKBP51 may act on the genetic liability to abnormal mood and anxiety states, it may provide a much needed treatment tool for secondary prophylaxis of depression recurrence and relapse.

## Materials and Methods

### Generation of *FKBP5−/−* mice

The mice have been generated as published previously [27]. Briefly, by PCR screening the 129SvJ mouse BAC library (Genome Systems, St. Louis, MO), bacterial artificial chromosome (BAC) clones that contained genomic regions for *FKBP5* were isolated. Restriction fragments were subcloned into pBluescript (pBS; Stratagene, La Jolla, CA) or pZero (Invitrogen, Carlsbad, CA) cloning vectors. The PCR products were amplified from the BAC clones and were then used to construct a targeting vector in the pPGK*neo* vector (a generous gift of James Lee, Mayo Clinic Scottsdale). The targeting vector contained a beta-galactosidase/neomycin cassette flanked by regions homologous to the *FKBP5* gene. Due to the size of the protein it is more practical to partially delete the gene. Thus, when the targeting vector integrates into the chromosome through homologous recombination it removes all of exon 2, which is the first coding exon. Since the only deleted portion of the gene is exon 2 the expression of the beta-galactosidase protein is dependent on the FKBP5 promoter and transcription machinery and expresses in frame with the initiation codon. ES cells were isolated from the 129SvJ mouse and cultured in Knockout DMEM media (Invitrogen) supplemented with 10% FBS, penicillin/streptomycin, essential amino acids, and ESGRO (103 U/ml; Chemicon, Temecula, CA) with irradiated embryonic fibroblast feeder cells. The ES cells were then electroporated at 0.2 kV, 950 µF (Gene Pulser II; Bio-Rad, Hercules, CA) with linearized targeting vectors and selected with G418. DNA from G418-resistant clones was isolated for Southern blot analysis. A DNA probe was used to distinguish *Pst*I restriction fragments from wildtype allele (∼7.5 kb) and targeting vector (∼10 kb). Appropriate homologous recombination in ES cell clones was confirmed by PCR using primers complementary to sequences within the neomycin cassette and to 3′ *FKBP5* sequences downstream from the recombination site. ES cell clones containing the targeting vector were injected into C57BL/6 blastocysts and implanted into pseudopregnant 129SvJ females. Chimeric offspring were identified by coat patterns and mated to C57BL/6 mice to obtain germline transmission of the targeting vector. For colony maintenance mice were crossed from C57BL6 onto Swiss-Webster for purposes of fecundity and genetic diversity to be more representative of a human population.

### Brain Tissue Fractionation and Western Blot Analysis

Brain tissue fractionation and western blot analysis were done as previously described [28].

### PCR

mRNA was isolated and purified from the brain of four wildtype and four *FKBP5−/−* mice using RNAeasy kit (QIAGEN, Valencia, CA). cDNA was synthesized from isolated mRNA by reverse transcription using Super Script III First-Strand cDNA Sythesis Kit (Invitrogen, Carlsbad, CA) from 50 ng of isolated mRNA. PCR was performed with synthesized cDNA and *FKBP5* specific primers to confirm presence or absence of *FKBP5* gene.

### Antibodies

Horseradish peroxidase conjugated secondary antibodies (Southern Biotech, Birmingham, AL), Glyceraldehyde-3-phosphate dehydrogenase antibody (Meridian Life Science, Saco, ME), Anti-FKBP51 was provided by Drs. David F. Smith and Marc Cox (Mayo Clinic, Scottsdale, AZ).

### Immunohistochemistry

Fixed mouse brains were processed for sectioning as previously described [29]. β-galactosidase staining was performed using the in situ β-gal staining kit (Stratagene, La Jolla, CA).

### Behavior

N = 9 unless otherwise noted. Video tracking software was used in several tests (ANY-Maze, Stoelting, Illinois).

### Open Field

Animals were monitored for 15 min in an open field with video tracking software.

### Rotorod test

Testing started at an initial rotation of 4 rpm accelerating to 40 rpm over 5 min. Mice were tested for 4 trials per day, for 2 consecutive days with a 30-min intertrial interval. Latency to fall from the rod onto a spring-cushioned lever was measured.

### Morris water maze (MWM)

Mice were trained to locate an escape platform hidden beneath the water (3 centimeter). Each mouse was given 4 trials per day with an intertrial interval of 1 hour for 6 consecutive days. Each animal was given 60 seconds to find the platform. Afterwards the mice were placed on the platform for 30 s. On day 7, mice were subjected to a trial in which the platform was removed, and had 60 s to search for it.

### Associative Fear Conditioning

Two mild foot shocks (0.5 milliamps) were paired with an auditory conditioned stimulus (CS, white noise, 70 decibels) within a novel environment. The CS was given for 30 s before each foot shock (2 s). Twenty-four hours later, the mice were placed in the chamber and monitored for freezing for 3 min (no shocks or CS). Immediately after the test, mice were placed into a novel context for 3 min without CS and then exposed to the CS for 3 min (cued).

### Prepulse Inhibition (PPI)

Mice were placed in a restrainer (Panlab, Barcelona Spain) and placed inside a sound attenuation chamber. The test consisted of 7 trial types in pseudorandom order: 1) 40 ms, 120 decibels sound burst (startle); 2–6) 5 different acoustic prepulses 100 ms in length, a 20 ms duration at 74, 78, 82, 86, and 90 dB; 7) no stimulus for baseline measurement. The intertrial interval was 15 s. The startle response peak was measured within a second after the stimulus.

### Elevated Plus Maze (EPM)

EPM consisted of 2 open arms facing each other and two enclosed arms also facing each other. Each arm is attached to the center platform and elevated 40 centimeters off the floor. The mouse was placed on the platform and allowed to explore for 5 min. Video tracking software measured movement.

### Porsolt forced swim test (FST)

Each mouse was placed in a 45 centimeters high and 20 centimeters diameter clear Plexiglas cylinder filled with room temperature water to a depth of 12 centimeters for 6 min. Amount of time spent immobile was recorded.

### Tail Suspension Test (TST)

Mice were suspended from their tail for 6 min. Amount of time spent immobile was recorded.

### Novel Object

Mice were placed in an area with two objects similar in scale to the mouse. Each animal was given 3 acclimation trials of 5 min with a 5-min intertrial interval. Then one acclimated object was replaced with a novel object. Animals were given a 5-min exploratory trial monitored by video recording.

### Y-maze

Animals were started at the center of the Y and allowed to explore for 8 min. Each session was video-monitored. The number of arm entries was recorded. The percent of spontaneous alternation was calculated as the number of triads containing entries into all three arms divided by the maximum possible of alternations (total number of entries minus 2).

### Corticosterone Assay, blood collection and stress paradigm

The levels of corticosterone were measured using an ELISA kit (Enzo Life Sciences, Plymouth Meeting, PA). Blood from mice was collected in the morning one hour after the light cycle began and 30 min after a 10-min tube restraint using the submandibular vein puncture method.

### Statistics

The student's t-test was used to compare 2 groups. The paired t-test was used to compare paired observations within 2 groups. The 2-way RMANOVA was used to compare the interaction between two dependent variables and an independent variable. The Bonferroni post test was used to correct for multiple testing. All error bars represent S.EM.

### Animal Study Approval

All animal procedures were approved by the University of South Florida Institutional Animal Care and Use Committee (IACUC), protocol #R3848.
